# Effect of pulmonary arteriovenous malformations on the mechanical properties of the lungs

**DOI:** 10.1186/s12890-017-0411-9

**Published:** 2017-04-19

**Authors:** Cécile Rotenberg, Marcel Bonay, Mostafa El Hajjam, Sandra Blivet, Alain Beauchet, Pascal Lacombe, Thierry Chinet

**Affiliations:** Consultation Pluridisciplinaire Maladie de Rendu Osler, Université de Versailles SQY, APHP, Hôpital Ambroise Paré, Boulogne-Billancourt, France

**Keywords:** Pulmonary arteriovenous malformation, Lung function, Hereditary hemorrhagic telangiectasia

## Abstract

**Background:**

Pulmonary arteriovenous malformations (PAVMs) are present in approximately 15–50% individuals with hereditary hemorrhagic telangiectasia (HHT). They may be isolated but more often are multiple. The goal of this study was to evaluate the influence of PAVMs on lung mechanical properties.

**Methods:**

We reviewed the files of all adult patients (age ≥ 18 years) referred to our Center for evaluation of HHT between 2005 and 2013. The diagnosis of HHT was based on the Curacao criteria and/or the presence of a pathogenic mutation. Exclusion criteria included: chronic cardiac or lung disease (i.e. asthma or COPD), suspicion of pulmonary hypertension on echocardiography, current or past smoking (>10 pack-years), history of thoracic surgery, previous treatment of PAVMs by embolotherapy, lung infection or thromboembolic disease in the past 3 months, pregnancy and obesity (BMI > 30 kg/m^2^). Chest high resolution CT-scan and pulmonary function tests were performed the same day in all patients as part of our routine work-up.

**Results:**

One hundred and fifty five patients with HHT were included (age: 44.4 ± 16.7 yrs – mean ± SD -; males: 39%). Eighty eight patients had no PAVM, 45 had 1–3 PAVMS and 22 had at least 4 PAVMs. Thirty eight patients had unilateral PAVMs and 29 bilateral PAVMs. We found no statistical relationship between the number, the size and the laterality of PAVMs and results of lung flows and volumes.

**Conclusion:**

We found no evidence that PAVMs have a significant influence on lung mechanical properties as measured using routine pulmonary function tests in adult patients with HHT, even in case of numerous, macroscopic or bilateral malformations.

## Background

Pulmonary arteriovenous malformations (PAVMs) are abnormal structures that connect the pulmonary arterial circulation to the pulmonary venous circulation without a capillary network, resulting in right-to-left shunt (RLS) [1, 2]. More than 80% of PAVMs are associated with an autosomal dominant genetic disease, hereditary hemorrhagic telangiectasia (HHT). HHT affects approximately 1–5/8000 people. HHT is caused by mutations in the endoglin (*ENG*) gene, the activin A receptor type II-like 1 (*ACVRL1/ALK1)* gene, and rarely, the smad4 gene [3]. PAVMs are found in 15% to 50% of HHT patients; 40% to 75% patients have *ENG* mutations and 5% to 45% patients have *ACVRL1/ALK1* mutations. These vascular malformations expose patients to severe complications due to paradoxical systemic emboli of thrombotic or septic origin, such as stroke and brain abscess [1–5]. PAVMs may be single or multiple, and unilateral or bilateral [1]. Although the natural history of PAVMs is not well known, evidence shows that a significant proportion may grow during follow-up [1, 2, 6].

Patients with PAVMs often complain of dyspnea [1, 7, 8]. This is related, at least in part, to the presence of RLS. We hypothesized that the presence of PAVMs may also impair the respiratory mechanical properties in relation to the number and/or size of PAVMs and therefore contribute to dyspnea.

Previous studies have reported the results of pulmonary function tests (PFTs) in patients with HHT and PAVMs [7, 9–12]. Most patients had normal resting pulmonary function values, while others showed a restrictive or an obstructive pattern (9% and 15%, respectively, in the largest series). However, patients were not selected according to the presence or absence of chronic heart or pulmonary diseases or previous lung surgery or embolization. Therefore, whether the reported abnormalities were related to PAVMs or to other conditions is unclear. Furthermore, no study has attempted to correlate the number or size of PAVMs with lung function values. We therefore decided to evaluate the influence of PAVMs on lung mechanical properties as measured using routine PFTs.

## Methods

### Study participants

We retrospectively reviewed the files of all consecutive adult patients with HHT who were referred to our center for evaluation between 2005 and 2013. The diagnosis of HHT was based on Curacao’s criteria and/or the presence of a pathogenic mutation [3]. Exclusion criteria included the following: age < 18 years, chronic cardiac or lung disease (such as asthma or COPD), suspicion of pulmonary hypertension on echocardiography, lung infection or thromboembolic disease in the past 3 months, current or past smoking (>10 pack-years), history of thoracic surgery, history of embolotherapy of PAVMs, pregnancy, and obesity (body mass index > 30 kg/m^2^). All patients underwent our routine evaluation protocol during the same day. This protocol usually included high resolution chest computed tomography, contrast echocardiography, PFTs, and genetic testing.

PFTs included measurements of total lung capacity (TLC), vital capacity (VC), forced expiratory volume in 1 s (FEV_1_), residual volume (RV), and forced expiratory flow at 75% of the vital capacity (FEF_75_). Lung function values that were corrected for sex, age, and height were used. We also measured the alveolar–arterial gradient in O_2_. Arterial blood sampling was performed with patients at rest in the sitting position.

Institutional review board approval was obtained for this retrospective study (CEPRO 2016–031).

### Statistical analysis

Quantitative data are expressed as mean ± standard deviation (SD) and qualitative data as frequency and percentage. Comparisons of two means were performed using the Student’s *t* test. Comparisons of three means were performed using one-way ANOVA. ANOVA was completed in case of significance by multiple comparisons tests. Comparisons of frequencies were performed using the chi-squared test. A *p* value < 0.05 was considered statistically significant. Statistical analysis was performed using R software version 3.0.2 (R Foundation for Statistical Computing, Vienna, Austria).

## Results

A total of 155 patients were included. Their characteristics are displayed in Table 1. Their mean age was 44.4 ± 16.7 years and 60 (39%) were men. Eighty-five (54.8%) patients had *ACVRL1/ALK1* mutations, 56 (36.1%) had *ENG* mutations, two (1.3%) had *smad4* mutations, and no mutation was identified in 12 (7.7%) patients. Sixty-seven (43.2%) patients had PAVMs visible on chest CT. PAVMs were more frequent in patients with *ENG* mutations than in those with *ACVRL1/ALK1* mutations (66.1% vs 30.6%, *p* < 0.001).

**Table 1:** Characteristics of the study population (*n* = 155)

AGE (years)	44.4 ± 16.7
MALE SEX (%)	60 (38.7%)
MUTATION	
ENG	56 (36.1%)
ACVRL1/ALK1	85 (54.8%)
Smad 4	2 (1.3%)
No mutation found	12 (7.7%)
PAVMs	
None	88 (56.8%)
1-3 PAVMs	45 (29.0%)
≥ 4 PAVMs	22 (14.2%)
TLC (% predicted)	99.3 ± 11.8 (*n* = 151)
RV (% predicted)	102.5 ± 26.0 (*n* = 151)
VC (% predicted)	102.1 ± 13.3 (*n* = 155)
FEV_1_ (% predicted)	101.6 ± 12.9 (*n* = 155)
FEV_1_/VC (%)	83.8 ± 11.8 (*n* = 155)
FEF_75_ (% predicted)	79.8 ± 29.5 (*n* = 154)
A-a O_2_ gradient (mmHg)	12.4 ± 10.9 (*n* = 151)

(PAVM = pulmonary arteriovenous malformation; TLC = total lung capacity; RV = residual volume; VC = vital capacity; FEV_1_ = forced expiratory volume in 1 s; FEF_75_ = forced expiratory flow at 75% of vital capacity; A-a O_2_ gradient = alveolar–arterial gradient in O_2_)

There were no significant associations between the presence of PAVMs on chest CT and TLC, RV, VC, FEV_1_, and FEF_75_. In contrast, the alveolar–arterial gradient in O_2_ was larger in patients with PAVMs (14.9 ± 13.2 mmHg) than in those without PAVMs (10.4 ± 8.2 mmHg, *p* < 0.05).

To study the effect of the number of PAVMs on lung function, we divided the patients into three groups: no PAVM visible on chest CT (*n* = 88, 56.8%), one to three PAVMs (*n* = 45, 29.0%), and ≥ four PAVMs (*n* = 22, 14.2%). Age and sex were similar among the three groups. We found no differences in lung function measurements among the groups, except for the alveolar–arterial gradient in O_2_, which was larger in patients with PAVMs than without (*p* < 0.01; Table 2).

**Table 2:** Comparison of lung function data between groups of patients according to the number, the size or the uni- or bilateral localization of the PAVMs

	TLC (% pred.)	RV (% pred.)	VC (% pred.)	FEV_1_ (% pred.)	FEF_25_ (% pred.)	A-a O_2_ gradient (mmHg)
Number						
No PAVM	98.7 ± 11.7 (*n* = 86)	102.9 ± 26.1 (*n* = 86)	101.7 ± 12.4 (*n* = 88)	101.4 ± 12.3 (*n* = 88)	80.4 ± 27.8 (*n* = 87)	10.4 ± 8.2 (*n* = 84)
1-3 PAVMs	99.4 ± 11.9 (*n* = 45)	99.0 ± 24.7 (*n* = 45)	103.8 ± 14.0 (*n* = 45)	102.9 ± 12.1 (*n* = 45)	79.1 ± 30.4 (*n* = 45)	13.3 ± 12.0 (*n* = 45)
≥ 4 PAVMS	101.9 ± 11.8 (*n* = 20)	108.7 ± 28.7 (*n* = 20)	100.4 ± 15.5 (*n* = 22)	99.7 ± 16.8 (*n* = 22)	78.7 ± 35.2 (*n* = 22)	18.2 ± 15.3 (*n* = 22)
p	NS	NS	NS	NS	NS	0.009
Size						
No PAVM	98.7 ± 11.7 (*n* = 86)	102.9 ± 26.1 (*n* = 86)	101.7 ± 12.4 (*n* = 88)	101.4 ± 12.3 (*n* = 88)	80.4 ± 27.8 (*n* = 87)	10.4 ± 8.2 (*n* = 84)
Microscopic PAVM(s)	100.7 ± 11.5 (*n* = 45)	97.1 ± 23.7 (*n* = 45)	104.5 ± 13.1 (*n* = 47)	103.4 ± 12.7 (*n* = 47)	82.2 ± 31.2 (*n* = 47)	10.9 ± 9.2 (*n* = 47)
≥ 1 macroscopic PAVM	99.0 ± 12.7 (*n* = 20)	113.0 ± 28.7 (*n* = 20)	98.3 ± 16.8 (*n* = 20)	98.0 ± 15.8 (*n* = 20)	71.5 ± 32.6 (*n* = 20)	24.5 ± 16.3 (*n* = 20)
p	NS	NS	NS	NS	NS	0.0001
Uni- or bilateral						
No PAVM	98.7 ± 11.7 (*n* = 86)	102.9 ± 26.1 (*n* = 86)	101.7 ± 12.4 (*n* = 88)	101.4 ± 12.3 (*n* = 88)	80.4 ± 27.8 (*n* = 87)	10.4 ± 8.2 (*n* = 84)
Unilateral PAVM(s)	100.1 ± 12.2 (*n* = 38)	101.8 ± 25.0 (*n* = 38)	102.7 ± 13.1 (*n* = 38)	102.7 ± 12.3 (*n* = 38)	80.4 ± 29.4 (*n* = 38)	11.9 ± 10.0 (*n* = 38)
Bilateral PAVMs	100.2 ± 11.5 (*n* = 27)	102.3 ± 28.3 (*n* = 27)	102.6 ± 16.4 (*n* = 29)	100.7 ± 15.6 (*n* = 29)	77.2 ± 35.1 (*n* = 29)	18.9 ± 15.9 (*n* = 29)
p	NS	NS	NS	NS	NS	0.001

(PAVM = pulmonary arteriovenous malformation; TLC = total lung capacity; RV = residual volume; VC = vital capacity; FEV_1_ = forced expiratory volume in 1 s; FEF_75_ = forced expiratory flow at 75% of vital capacity; A-a O_2_ gradient = alveolar–arterial gradient in O_2_. NS = not significant)

To study the effect of size of the PAVMs on lung function, we compared functional data in patients with no PAVMs, those with microscopic PAVMs (defined by a feeding artery with a diameter < 2.5 mm), and those with at least one macroscopic PAVM (defined by a feeding artery with a diameter ≥ 2.5 mm and thus amenable to embolization). Age and sex were similar among the three groups. We found no difference in lung function measurements among the groups, except for the alveolar–arterial gradient in O_2_, which was larger in patients with macroscopic PAVMs compared with patients without PAVM and with only microscopic PAVMs (*p* < 0.0001 for all comparisons; Table 2).

We also compared functional data in patients with no PAVMs, those with unilateral PAVM(s), and those with bilateral PAVMs. Age and sex were similar among the three groups. We found no difference in lung function measurements among the groups, except for the alveolar–arterial gradient in O_2,_ which was larger in patients with bilateral PAVMs compared with patients without PAVM (*p* < 0.01) and with only unilateral PAVM(s) (*p* < 0.05) (Table 2).

## Discussion

To the best of our knowledge, our study is the largest to evaluate the effect of PAVMs on lung mechanical properties. Our main finding is the absence of an effect of PAVMs, even in patients with numerous and/or bilateral PAVMs. As expected, there was a significant relationship between the alveolar–arterial O_2_ gradient, and the number and size of PAVMs.

Vascular disease may affect lung mechanics. Previous studies on pulmonary function in patients with pulmonary arterial hypertension (PAH) have reported a restrictive defect [13] or reduced expiratory flow at low lung volumes [14]. This flow limitation may be present in up to 60% of patients with PAH. Another study showed that, in young nonsmoking patients with PAH, reduced expiratory flow at low lung volumes was associated with dynamic hyperinflation in response to an exercise-related increase in ventilation [15]. This finding suggests that abnormal ventilatory mechanics may contribute to the genesis of dyspnea in these patients. Therefore, in our series, we did not include patients with PAVMs and signs of PAH on echocardiography. Our results differ from those in patients with PAH.

Dyspnea in patients with PAVMs is considered to mainly result from hypoxemia caused by right-to-left shunting [1]. Other causes of dyspnea in patients with PAVMs may include anemia, PAH, and high-output cardiac failure resulting from diffuse liver vascular malformations. Our results suggest that PAVMs do not contribute to dyspnea through alteration of lung mechanics.

This study has several limitations: first, it was a single-center, retrospective study. Second, we focused only on resting pulmonary function and did not attempt to measure lung function during exercise. Third, PFTs values are insensitive to small changes in lung mechanics, especially small airways. However, small changes in lung mechanics are unlikely to have a significant clinical impact in these patients. Finally, we did not include a non-HHT control group to determine the impact of HHT itself (without detectable PAVM) on lung mechanics.

## Conclusions

In conclusion, we found no association between the presence of PAVMs in patients with HHT and mechanical properties of the lung as evaluated using routine PFTs, even in patients with numerous, macroscopic or bilateral malformations, suggesting that the presence of PAVMs does not contribute to the patients’dyspnea by a mechanical effect.

## Abbreviations

ACVRL1/ALK1: Activin A receptor type II-like 1
ANOVA: Analysis of variance
CT: Computed tomography
ENG: Endoglin
FEF_75_: Forced expiratory flow at 75% of the vital capacity
FEV_1_: Forced expiratory volume in one second
HHT: Hereditary haemorrhagic telangiectasia
PAH: Pulmonary arterial hypertension
PAVM: Pulmonary arteriovenous malformations
PFTs: Pulmonary function tests
RLS: Right-to-left shunt
RV: Residual volume
SD: Standard deviation
TLC: Total lung capacity
VC: Vital capacity
